# Carbon Nanomaterials for Sorption of ^68^Ga for Potential Using in Positron Emission Tomography

**DOI:** 10.3390/nano10061090

**Published:** 2020-06-01

**Authors:** Andrey G. Kazakov, Bogdan L. Garashchenko, Milana K. Ivanova, Sergey E. Vinokurov, Boris F. Myasoedov

**Affiliations:** 1 Vernadsky Institute of Geochemistry and Analytical Chemistry of the Russian Academy of Sciences, Radiochemistry Laboratory, 19 Kosygin St., Moscow 119991, Russia; bogdan@garashchenko.com (B.L.G.); milaprezident@mail.ru (M.K.I.); vinokurov.geokhi@gmail.com (S.E.V.); bfmyas@mail.ru (B.F.M.); 2 Interdepartmental Center for Analytical Research on Problems in the Field of Physics, Chemistry, and Biology, of the Russian Academy of Sciences, Bld. 6, Profsoyuznaya St. 65, Moscow 117342, Russia

**Keywords:** carbon nanomaterials (CNMs), ^68^Ga, sorption and desorption, surface functionalization, nanodiamond (ND), reduced graphite oxide (rGiO), multi-walled carbon nanotubes (MWCNT), positron–emission tomography (PET)

## Abstract

In present work, carbon nanomaterials (CNMs) are investigated as potential carriers of ^68^Ga, which is widely used in positron emission tomography (PET) in nuclear medicine. Sorption behavior of ^68^Ga was studied onto CNMs of various structures and chemical compositions: nanodiamonds (ND), reduced graphite oxide (rGiO) and multi-walled carbon nanotubes (MWCNT), as well as their oxidized (ND–COOH) or reduced (rGiO–H, MWCNT–H) forms. The physicochemical properties of the nanoparticles were determined by high resolution transmission electron microscopy, x-ray photoelectron spectroscopy, dynamic light scattering and potentiometric titration. The content of ^68^Ga in the solutions during the study of sorption was determined by gamma-ray spectrometry. The highest degree of ^68^Ga sorption was observed on ND and ND–COOH samples, and the optimal sorption conditions were determined: an aqueous solution with a pH of 5–7, m/V ratio of 50 μg/mL and a room temperature (25 °C). The ^68^Ga@ND and ^68^Ga@ND–COOH conjugates were found to be stable in a model blood solution—phosphate-buffered saline with a pH of 7.3, containing 40 g/L of bovine serum albumin: ^68^Ga desorption from these samples in 90 minutes was no more than 20% at 25 °C and up to 30% at 37 °C. Such a quantity of desorbed ^68^Ga does not harm the body and does not interfere with the PET imaging process. Thus, ND and ND–COOH are promising CNMs for using as carriers of ^68^Ga for PET diagnostics.

## 1. Introduction

Nowadays, nanomaterials, including carbon nanomaterials (CNMs), are considered as promising carriers of bonded with them radionuclides for nuclear medicine, including positron emission tomography (PET) diagnostic, due to their physicochemical properties. CNMs range in size from a few nm to hundreds of nm, which is comparable to biologic macromolecules (including proteins, enzymes and plasmid DNA) [1], so CNMs can penetrate cell membranes [2], which allows them to be used as drug carriers [3]. Size of CNMs also allow targeted delivery of radionuclides to the affected organ or tissue, penetrate and selectively accumulate in the tumor at much higher concentrations than in the surrounding healthy tissue [4,5], and also be retained due to the abnormally developed capillary network and slow outflow from the lymphatic system. The development of radiopharmaceuticals (RPs) based on using nanoparticles, with different radionuclide bonded with them (including using sorption process) continues to attract the attention of a growing number of researchers [6,7,8,9,10,11].

For targeted delivery of various nuclear medicine radionuclides in general [12,13,14,15,16,17,18] and of PET radionuclides in particular [19,20], such promising CNMs as nanodiamond (ND), graphene oxide, its derivatives and carbon nanotubes, are considered as carriers. PET (a field of nuclear medicine) is a method for visualization of the internal organs of an animal or person using drugs containing a positron-emitting radionuclide, based on the detection of a pair of gamma rays arising from the annihilation of positrons with electrons [21]. PET has been used for decades to detect and visualize cancerous tumors in the body [22]. As an example of researches of CNMs using in PET, one can distinguish ND as carrier of widely used PET radionuclide ^18^F [19] or single-walled carbon nanotubes, which were found to be an effective carriers for perspective PET radionuclide ^89^Zr [20].

Among radionuclides suitable for PET, ^68^Ga is one of the most promising isotopes for widespread use [23,24,25]. It has a suitable set of nuclear physical characteristics (T_1/2_ = 68 min, 100% β^+^) and high availability, as it can be obtained from a ^68^Ge/^68^Ga generator. The prospect of studying new nanoparticles for the targeted delivery of ^68^Ga into the human body is noted in works [6,26] where nanoparticles based on polysiloxane matrix and nanoparticles of carbon were used for these purposes.

In our recent work [27] we demonstrated the ability of different CNMs to sorb nuclear medicine isotopes of Tc(IV,VII), Bi(III), Y(III). The main aim of the present work was to study the optimal conditions of sorption of ^68^Ga onto CNMs of different structure and composition depending on various factors, as well as the study of the stability of the obtained conjugates in biologic media, for their further possible applications in PET diagnostics.

## 2. Materials and Methods

### 2.1. CNMs Preparation and Characterization

Commercial samples of the following CNMs were used: ND powder (SKTB Technolog, Saint-Petersburg, Russia, trademark UDA-TAN), reduced graphite oxide (rGiO) aqueous suspension and multi-walled carbon nanotubes (MWCNT) powder (NanoTekhTsentr Ltd, Tambov, Russia).

All chemical reagents used in the work had purity not lower than “chemically pure”.

Surface functionalization of initial CNMs (oxidation of ND and hydrogenation of rGiO and MWCNT) was conducted as follows.

To obtain a sample of oxidized ND (named ND–COOH), 1 g of initial ND was boiled in 75 mL of mixture of concentrated H_2_SO_4_ and HNO_3_ (3:1 by volume) for 24 hours at 120 °C with constant stirring (reaction 1). The resulting ND–COOH sample was separated from acid solution by centrifugation at 15,000 g for 15 min (CM-50, Eppendorf, Hauppauge, NY, USA; centrifugation was always used for every separation step described further), then twice washed by 50 mL of 0.1-M NaOH to remove acids residue and, finally, washed by double distilled water up to its pH was about 7. After all purification steps, ND–COOH bulk finally was dried on a rotary evaporator at 60 °C.

(1) $$\mathrm{ND}\;\overset{H_{2}{\mathrm{SO}}_{4}/{\mathrm{HNO}}_{3}\;\left(3:1\right),\;\left(120\;˚C,\;24\;h\right)}{\to }\;\mathrm{ND}–\mathrm{COOH}$$

To synthesize a sample of hydrogenated rGiO (rGiO–H), the aqueous suspension of rGiO was dried. A portion of the dried rGiO was poured into a boat and placed in a quartz reactor. Gas mixture containing 10% of 99.99% hydrogen and 90% of 99.9% Ar was then introduced into the cold reactor for 20 min to remove air from the system. The gas mixture flow rate (about 2–3 L/h) was measured using a foam flow meter. After that the reactor was placed in a furnace and the reaction was carried out for 5 h at 800 °C (reaction 2). Then, the obtained rGiO–H sample was kept in a stream of a gas mixture until it was completely cooled. MWCNT were hydrogenated under the same conditions, but without preliminary drying that resulted in the formation of a sample MWCNT–H.

(2) $$\mathrm{rGiO},\;\mathrm{MWCNT}\;\overset{10{\%\;H}_{2}+90\%\;\mathrm{Ar}\;\left(800\;˚C,\;5\;h\right)}{\to }\;\mathrm{rGiO}–H,\;\mathrm{MWCNT}–H$$

Earlier [27] we determined the physicochemical properties of commercial and modified CNMs samples used in this research. The physicochemical properties of the nanoparticles were determined by high resolution transmission electron microscopy (HRTEM) (JEOL JEM-2100F/Cs/GIF (200 kV, 0.8 A), Tokyo, Japan), X-ray photoelectron spectroscopy (XPS) (Kratos Axis Ultra DLD, Manchester, GM, UK), dynamic light scattering (DLS) (ZetaSizer Nano ZS analyzer (633 nm) ZEN 3600, Malvern, Worcestershire, UK) and potentiometric titration; Table 1 summarizes the main characteristics of the CNMs used and more detail information is presented in Appendix A. It is seen from Table 1 that CNMs significantly differed both in structure and composition. Thus, NDs are spherical particles, rGiOs are nanoparticles of a flat structure and MWCNTs have a micron-sized filamentary structure with an internal cavity. In addition, the results of XPS and titration show changes in the composition of the surface of the samples after reactions (1) and (2). Thus, the oxygen content for ND–COOH increases by 2.2% compared with the ND sample and the content of carboxyl groups is also increased three times, which indicates the successful completion of the oxidation reaction and makes possible a difference in the sorption properties of ND and ND–COOH. Moreover, the size of aggregates in hydrosols varies slightly. As for the reduction process of rGiO and MWCNT, the oxygen content on the rGiO–H surface becomes significantly lower—by 11.6% and for MWCNT–H it decreases by only 0.4%. At the same time, the size of the aggregates for both rGiO–H and MWCNT–H undergoes significant changes, which indicates the influence of the hydrogenation process on the structure and suggests some difference in sorption properties.

### 2.2. Radionuclide Separation and Detection

All experiments were conducted in a glove box.

No-carrier-added ^68^Ga isolated from a mother solution of ^68^Ge (produced by Cyclotron Co., Ltd., Obninsk, Russia) by liquid–liquid extraction in an 8 M HCl and CCl_4_ system (1:1) [28] was used in our experiments. The phases were intensively mixed for 2 min, after which they were separated, while ^68^Ge quantitatively passed into the organic phase and radiochemically pure ^68^Ga remained in the aqueous phase. The aqueous fraction of ^68^Ga was evaporated to dryness in a glass beaker.

The ^68^Ga content in the studied solutions was determined by gamma-ray spectrometry using the 1077 keV line on a spectrometer with a high-purity germanium detector GC 1020 (Canberra Ind, Meriden, CT, USA).

### 2.3. Sorption Experiments

To make the initial solutions of ^68^Ga to study its sorption, solution of HCl or NH_3_ was added to the dry residue obtained after evaporation to reach solutions with pH 1, 3, 5 or 7. Aliquots of these solutions and of suspension of CNMs were successively added to the Eppendorf tube, with a total volume of 1 mL. The kinetics of sorption and desorption of ^68^Ga in an aqueous solution was determined using suspensions containing from 10 to 200 μg CNMs. The experiments were carried out at 25 (sorption and desorption) and 37 °C (desorption); the temperature was controlled by a thermal shaker attachment (TS-100, Biosan, Latvia); rate of shaking was 1100 rpm. After sorption, the phases were separated by centrifugation for 15 min at 15,000 g (in preliminary experiments, it was shown that these conditions are sufficient for quantitative phase separation), 500 μL of the supernatant were taken and the gamma spectrum was registered, comparing with the initial activity of the solution.

The stability of the obtained ^68^Ga@CNMs conjugates was evaluated by studying desorption of ^68^Ga in a model blood solution—phosphate-buffered saline (PBS) with pH 7.3 containing 40 g/L bovine serum albumin (BSA). The solution in the test tube was agitated, placed on a shaker, then centrifuged again and the supernatant was separated for measurement.

The ^68^Ga activity in each sample was about 50 kBq, which was equivalent to a concentration of 5 × 10^−15^ M during our experiments. Each experiment with ^68^Ga sorption or desorption was carried out at least three times until converging values.

## 3. Results

The sorption of ^68^Ga onto ND, ND–COOH, rGiO, rGiO–H, MWCNT and MWCNT–H was studied in present work, as well as the stability of the obtained conjugates in model biologic media.

### 3.1. Selection of Optimal Conditions for ^68^Ga Sorption onto CNMs

It is well known that the efficiency of sorption of radionuclides on sorbents of various properties depends on the chemical properties of the sorbed element, the structure and physicochemical properties of the sorbent, as well as the ratio of the mass of the sorbent to the volume of the solution (m/V), its properties (including pH) and other factors.

#### 3.1.1. Kinetics of ^68^Ga Sorption by CNMs

**Figure 1:**
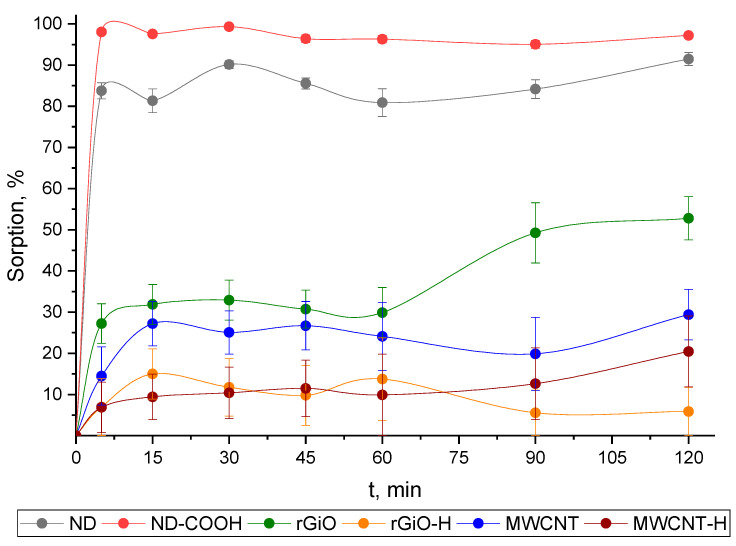
Sorption kinetics of ^68^Ga by studied CNMs from an aqueous solution with a pH 7 at m/V ratio of 100 μg/mL and 25 °C.

The kinetics of ^68^Ga sorption from aqueous solutions with pH 7 and m/V ratio of 100 μg/mL was studied on commercial CNMs (ND, rGiO and MWCNT) and their forms with surface modification, prepared in our experiments—oxidized ND (ND–COOH) and hydrogenated rGiO and MWCNT (rGiO–H and MWCNT–H); the results are presented in Figure 1. It was found that for ND and ND–COOH particles, sorption equilibrium is reached in 5 min and the degree of sorption of ^68^Ga is about 85 and not less than 95%, respectively. Sorption of ^68^Ga on the rGiO sample under studied conditions occurs more slowly than on ND and ND–COOH and sorption of only about 50% ^68^Ga is observed at 120 min, but sorption equilibrium is not achieved (Figure 1). On the samples rGiO–H, MWCNT and MWCNT–H, equilibrium is established within the first 5 min, but the maximum sorption of ^68^Ga does not exceed 30% for MWCNT and about 15% for rGiO–H and MWCNT–H in 120 min. Thus, of all CNMs studied in the work, only ND and ND–COOH have high sorption ability for ^68^Ga from aqueous solutions with pH 7.

#### 3.1.2. The Influence of the m/V Ratio to the Sorption Efficiency of ^68^Ga onto CNMs

**Figure 2:**
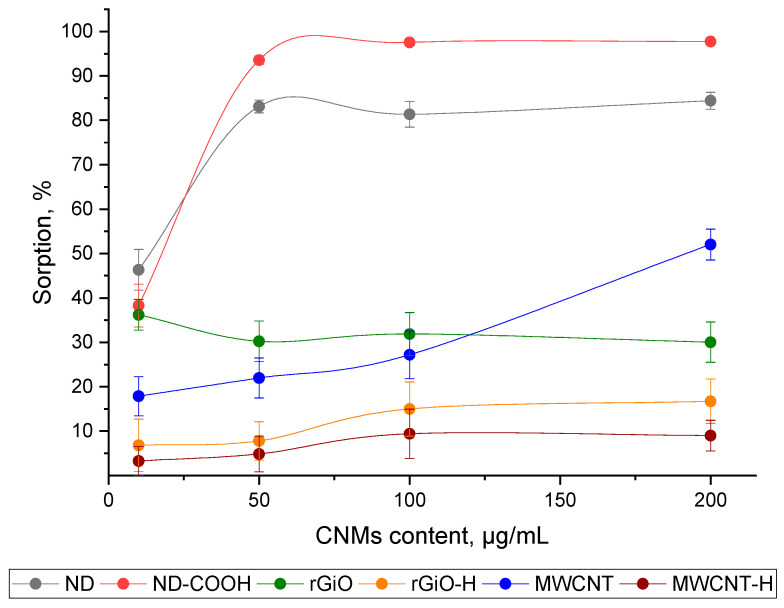
Sorption of ^68^Ga by studied CNMs at various m/V ratios from an aqueous solution with a pH 7 at 25 °C.

The sorption of ^68^Ga onto all studied CNMs samples from aqueous solutions with pH 7 at various m/V ratios was studied. As can be seen from the data obtained (Figure 2), the maximum sorption onto ND and ND–COOH samples was achieved at 50 μg/mL and changes slightly with a further increase in m/V to 200 μg/mL. The efficiency of sorption of ^68^Ga on MWCNT monotonously increases with an increase in m/V from 10 to 200 μg/mL and is at the maximum of about 50%. In the case of rGiO, rGiO–H and MWCNT–H, the maximum sorption of ^68^Ga was achieved at a ratio of 10 μg/mL and does not change much with an increase of up to 200 μg/mL.

#### 3.1.3. The Influence of pH of Initial Solution ^68^Ga on the Efficiency of its Sorption onto ND and ND–COOH

**Figure 3:**
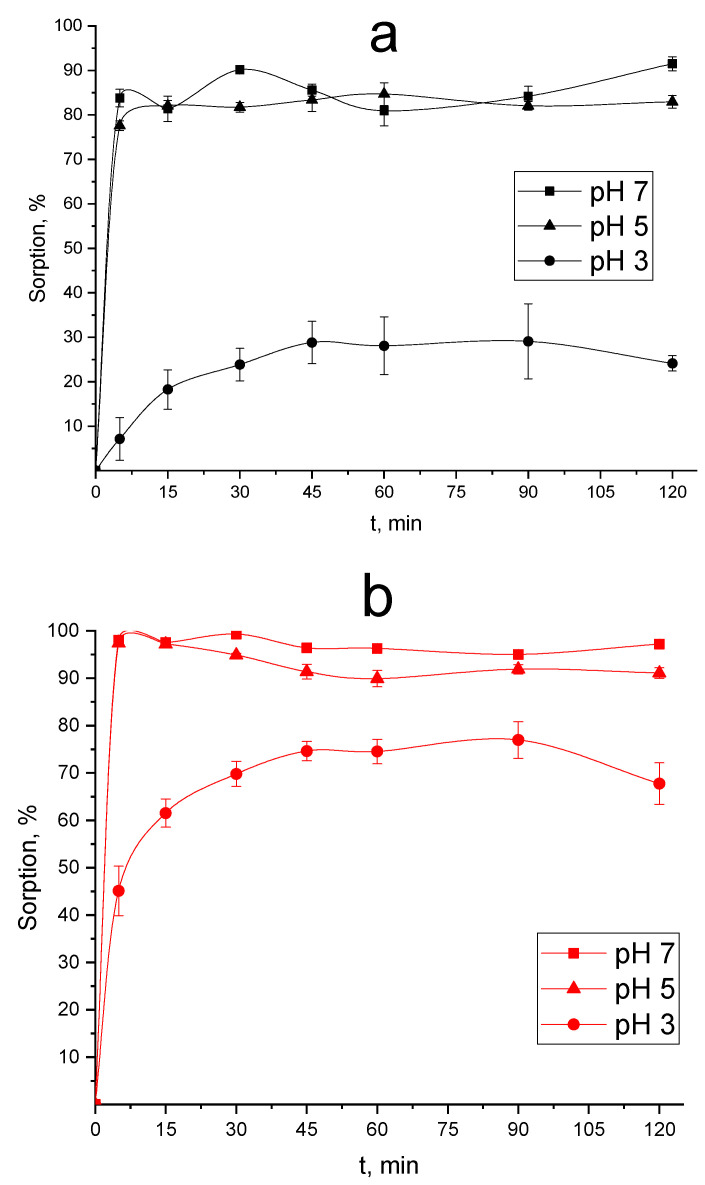
The influence of pH of the initial solution to the ^68^Ga sorption onto nanodiamonds (ND) (**a**) and their oxidized forms (ND–COOH) (**b**) at m/V ratio of 100 μg/mL and at 25 °C.

It was shown that ND and ND–COOH efficiently sorb ^68^Ga from a solution of pH 7 (Figure 1 and Figure 2) and then, the influence of pH of initial solution ^68^Ga on the efficiency of its sorption onto ND and ND–COOH was studied. The obtained data on the ^68^Ga sorption from aqueous solutions with pH 1, 3, 5 and 7 are shown in Figure 3. It was demonstrated that at pH 5 the degree of sorption of ^68^Ga for both CNMs samples decreased slightly (about 5%) compared to pH 7, while at pH 3 there was a significant decrease in sorption of ^68^Ga onto ND to 25% in 120 min and sorption onto ND–COOH also decreases—to 75% at the same time. In addition, sorption on both CNMs samples at pH 3 slows down and equilibrium is achieved by mixing the phases in 45 min, while at pH 5 and 7—in 5 min. At pH 1, as shown by our studies, ^68^Ga sorption is not observed on both samples for 120 min.

### 3.2. Study of the Stability of ^68^Ga@CNMs Conjugates in Model Biologic Media

**Figure 4:**
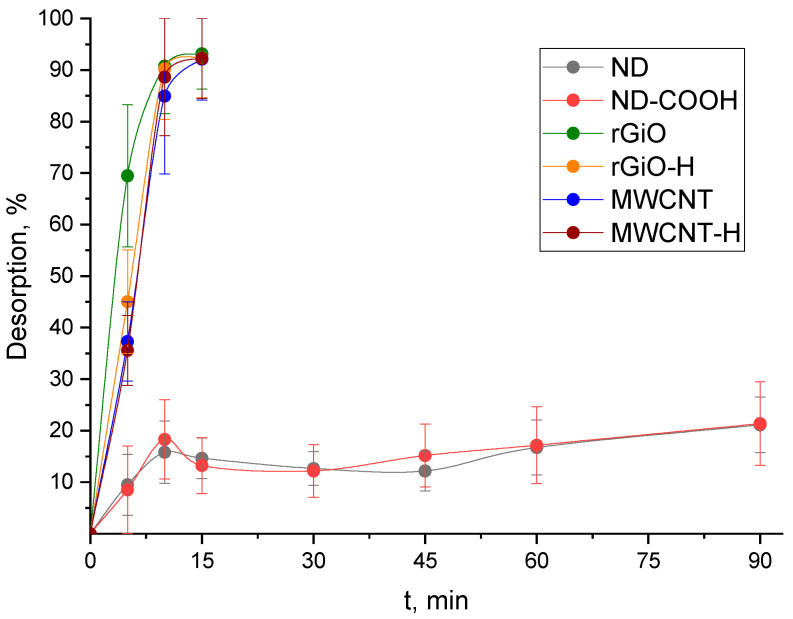
Desorption of ^68^Ga from studied CNMs (100 μg/mL) by the solution of 40 g/L bovine serum albumin (BSA) in phosphate-buffered saline (PBS) at 25 °C.

**Figure 5:**
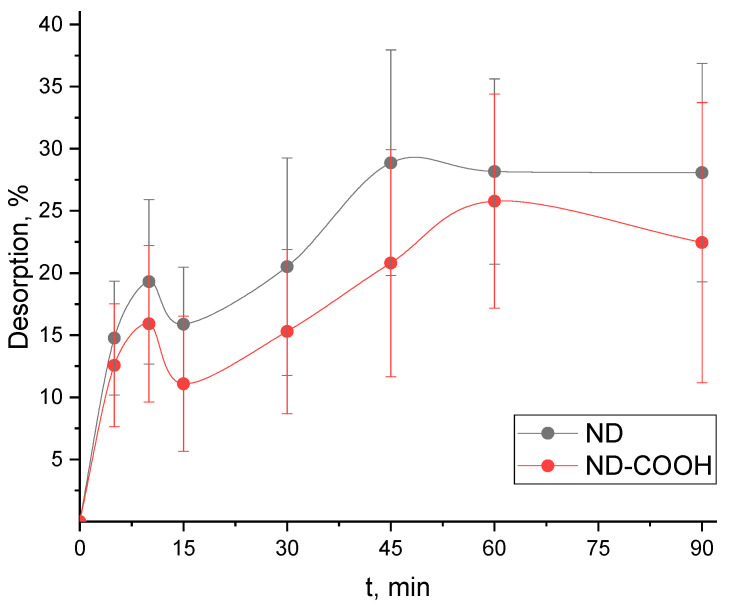
Desorption of ^68^Ga from ND and ND–COOH (100 μg/mL) by the solution of 40 g/L BSA in PBS at 37 °C.

It is known that the model solution of 40 g/L BSA in PBS, which simulates the salt composition, pH and the content of proteins in human blood, is most used to study the stability of the developed RPs [29,30,31]. We used this solution to study the stability of the developed ^68^Ga@CNMs conjugates at 25 and 37 °C (room and human body temperature; Figure 4 and Figure 5, respectively).

As can be seen from the data in Figure 4, the lowest degree of desorption of ^68^Ga is observed for ND and ND–COOH, which reaches 20% in 90 min and is acceptable for using developed conjugates (^68^Ga@CNMs). For rGiO, rGiO–H, MWCNT and MWCNT–H, quantitative desorption of ^68^Ga occurs in 15 min, which indicates the impossibility to use these CNMs as carriers of ^68^Ga in RPs. In this regard, the influence of temperature of 37 °C to stability was studied for ^68^Ga@ND and ^68^Ga@ND–COOH conjugates. As follows from the data in Figure 5, an increase in the temperature of the stripping solution from 25 to 37 °C leads to a change in the degree of desorption of the radionuclide from 20% to 30% in 90 min.

## 4. Discussion

The results of the study of ^68^Ga sorption (Figure 1) indicate the dominant role of carboxyl groups in its interaction with the studied CMNs. Thus, the maximum sorption values were achieved for ND and ND–COOH having carboxyl groups on the surface; the increase in ^68^Ga sorption on the ND–COOH sample compared to commercial ND is associated with an increase in the content of carboxyl groups on the ND–COOH surface from 330 to 990 μmol/g (as seen from the data in Table 1). In the case of rGiO, rGiO–H, MWCNT and MWCNT–H samples, a relatively low ^68^Ga sorption was observed even in a chemically inert aqueous solution, which, obviously, is associated with weak physical interactions of gallium cations of the solution with the surface of these CNMs.

When considering the data of study of the influence of the m/V ratio to the sorption efficiency of ^68^Ga onto CNMs (Figure 2), we can conclude that the optimal m/V ratio is 50 μg/mL, providing quantitative sorption of ^68^Ga and also reduce the possible negative impact of the carrier (ND or ND–COOH) on the organism.

The results of studying of the influence of pH of initial ^68^Ga solution on the efficiency of its sorption onto ND and ND–COOH (Figure 3) confirm the assumption on the main mechanism of sorption of ^68^Ga by ND and ND–COOH. From this point of view, the decrease in sorption following by acidity increasing can be explained by the fact that a strong acid in solution suppresses the dissociation of weak carboxyl groups and gallium cations cease to interact with their anions. Hence, the most promising CNMs among studied for the obtaining of conjugates with ^68^Ga are ND and ND–COOH and following optimal conditions at 25 °C: solutions with pH 5 to 7, m/V ratio 50 μg/mL and sorption time 5 min.

High degree of desorption of ^68^Ga from all studied CMNs except ND and ND–COOH (Figure 4) reaffirms the assumption about the role of carboxyl groups. It can be seen that samples containing no such groups (rGiO, rGiO–H, MWCNT, MWCNT–H) instantly release gallium in the protein medium in PBS, while ND and ND–COOH continue to hold them relatively firmly. Thus, we can conclude that there is a clear need for the presence of carboxyl groups on the surface of the ^68^Ga CMNs carriers to create stable conjugates. Temperature increasing to 37 °C leads to low increasing of ^68^Ga desorption from ND and ND–COOH (Figure 5) and such a percent of desorbed ^68^Ga does not harm the body. On the other hand, such an amount of ^68^Ga desorbed from the surface of CNMs during PET procedures may bind to blood proteins and cause significant noise. We believe that to solve this problem, an increase in the content of carboxyl groups on the surface of CNMs for stronger retention of ^68^Ga is required, as well as coating ^68^Ga@CNMs conjugates with biocompatible materials that would interfere with ^68^Ga desorption (for example, polyethylene glycol [32,33]). 

## 5. Conclusions

In the present work, sorption of ^68^Ga by various commercial CNMs (ND, rGiO and MWCNT) and their oxidized (ND–COOH) or reduced (rGiO–H and MWCNT–H) forms was studied. It was shown that ND and ND–COOH sorb ^68^Ga about 85% and 95% and more, respectively, while the rest of the samples (rGiO, rGiO–H, MWCNT, MWCNT–H) sorb not more than 50%. The optimal conditions for sorption of gallium on ND and ND–COOH were determined: solutions with pH five to seven, meters/V ratio 50 μg/mL and sorption time five minutes. It was also found that ^68^Ga desorption from ND and ND–COOH in a model biologic medium does not exceed 30% in 90 min, while quantitative desorption was observed from other studied CNMs in 15 min.

The choice of better carrier of ^68^Ga between ND and ND–COOH is not straightforward and depends on a number of factors. On one hand, ND–COOH sorbs 10% more ^68^Ga than ND and also slightly less ^68^Ga is desorbed from its surface than from the ND surface at the same time. On the other hand, ND oxidation procedure increases the cost of the ND–COOH sample and its use may turn out to be much less advantageous, although, as was shown in our work, only ratio of 50 μg ND–COOH per mL of solution is sufficient for quantitative sorption of ^68^Ga.

To conclude, the data obtained in the work indicate that both ^68^Ga@ND and ^68^Ga@ND–COOH conjugates are promising to found application in positron emission tomography, but further researches—especially including their stability in vivo—are required.

## Figures and Tables

**Table 1:** Characterization of carbon nanomaterials (CNMs).

Commercial (Initial) CNMs			
Characteristics	ND	rGiO	MWCNT
Particle size of the original samples (nm)	Spherical particles 3–10	Nanosheets—2 Sheets > 10^2^	Length > 2 × 10^4^ Diameter—30 Wall thickness 5–10
Elemental composition of the surface according to XPS,%	C (sp^3^)—92.3 O—7.7 N—1.0	C (sp^2^)—77.4 C (sp^3^)—7.9 O—14.7	C (sp^2^)—99.0 O—1.0
The size of particles and their aggregates in hydrosols, nm	100	Particles—2 Sheets—n/d^1^	n/d^1^
Amount of -COOH according to titration, μmol/g	330	–	–
**Modified CNMs**			
**Characteristics**	**ND–COOH**	**rGiO–H**	**MWCNT–H**
Elemental composition of the surface according to XPS,%	C(sp^3^)—89.1 O—9.9	C (sp^2^)—72.3 C (sp^3^)—24.6 O—3.1	C (sp^2^)—99.4 O—0.6
The size of particles and their aggregates in hydrosols, nm	95	200 and 700	150 and 650
Amount of -COOH according to titration, μmol/g	990	–	–

^1^ impossible to determine by dynamic light scattering if at least one of the linear particle sizes exceeds 1000 nm.

**Figure A1:**
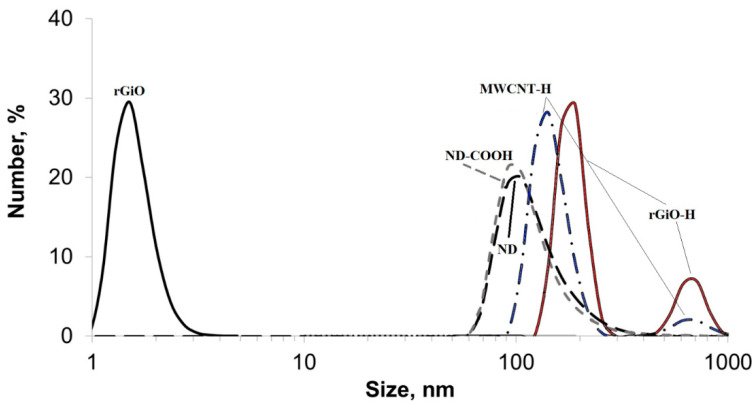
CNMs particle size distributions according to (DLS).

**Figure A2:**
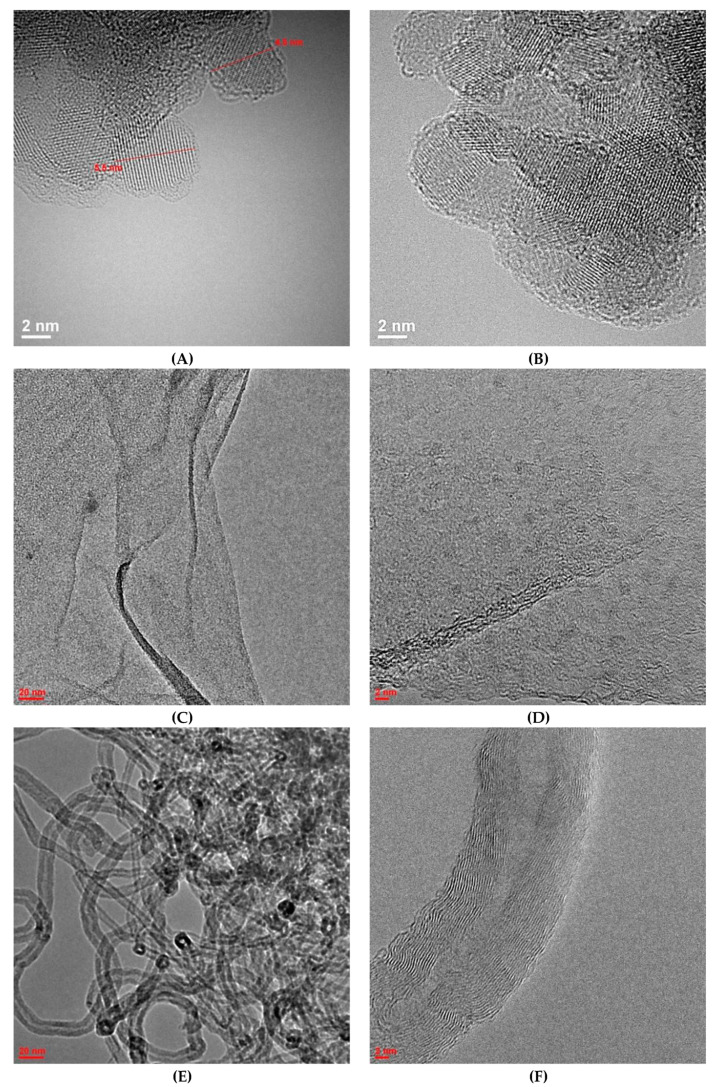
HRTEM images of commercial CNMs used: ND (**A**,**B**), rGiO (**C**,**D**), multi-walled carbon nanotubes (MWCNT) (**E**,**F**); images are similar to ones for modified samples ND–COOH, rGiO–H and MWCNT–H, respectively. Adapted from [Kazakov, A.G.; Garashchenko, B.L.; Yakovlev, R.Y.; Vinokurov, S.E.; Kalmykov, S.N.; Myasoedov, B.F. An experimental study of sorption/desorption of selected radionuclides on carbon nanomaterials: a quest for possible applications in future nuclear medicine. Diam. Relat. Mater. 2020, 104, 107752, doi:10.1016/j.diamond.2020.107752.] with permission from Elsevier B.V., 2020.

**Figure A3:**
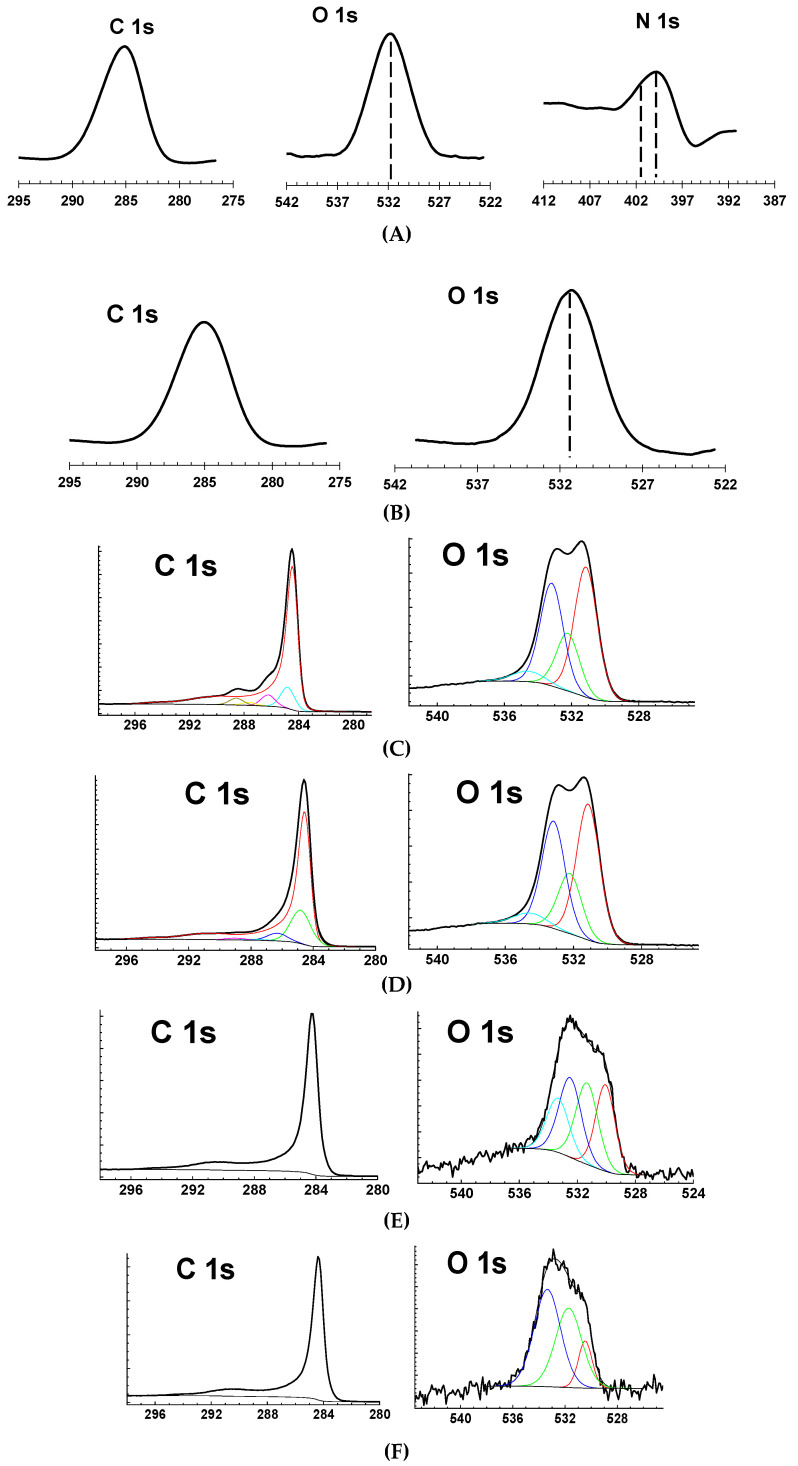
XPS images of CNMs used: ND (**A**), ND–COOH (**B**), rGiO (**C**), rGiO–H (**D**), MWCNT (**E**), MWCNT–H (**F**).

## References

[1] Yamashita T., Yamashita K., Nabeshi H., Yoshikawa T., Yoshioka Y., Tsunoda S., Tsutsumi Y. (2012). Carbon Nanomaterials: Efficacy and Safety for Nanomedicine. Materials.

[2] Hong G., Wu J.Z., Robinson J.T., Wang H., Zhang B., Dai H. (2012). Three-dimensional imaging of single nanotube molecule endocytosis on plasmonic substrates. Nat. Commun..

[3] Mendes R.G., Bachmatiuk A., Büchner B., Cuniberti G., Rümmeli M.H. (2013). Carbon nanostructures as multi-functional drug delivery platforms. J. Mater. Chem. B.

[4] Peer D., Karp J.M., Hong S., Farokhzad O.C., Margalit R., Langer R. (2007). Nanocarriers as an emerging platform for cancer therapy. Nat. Nanotechnol..

[5] Chow E.K., Zhang X.Q., Chen M., Lam R., Robinson E., Huang H., Schaffer D., Osawa E., Goga A., Ho D. (2011). Nanodiamond therapeutic delivery agents mediate enhanced chemoresistant tumor treatment. Sci. Transl. Med..

[6] Truillet C., Bouziotis P., Tsoukalas C., Brugière J., Martini M., Sancey L., Brichart T., Denat F., Boschetti F., Darbost U. (2015). Ultrasmall particles for Gd-MRI and 68 Ga-PET dual imaging. Contrast Media Mol. Imaging.

[7] Huclier-Markai S., Alliot C., Tillement O., Cutler C.S., Ntsiba E., Thomas E., Lux F. (2019). Multimodal AGuIX® Nanoparticles: Size Characterization by HF5 and Optimization of the Radiolabeling with Various SPECT/PET/Theranostic Tracers. Int. J. Med. Nano Res..

[8] Lamb J., Holland J.P. (2018). Advanced Methods for Radiolabeling Multimodality Nanomedicines for SPECT/MRI and PET/MRI. J. Nucl. Med..

[9] Thomas R., Park I.-K., Jeong Y. (2013). Magnetic Iron Oxide Nanoparticles for Multimodal Imaging and Therapy of Cancer. Int. J. Mol. Sci..

[10] Xie Z., Su Y., Kim G.B., Selvi E., Ma C., Aragon-Sanabria V., Hsieh J.-T., Dong C., Yang J. (2017). Immune Cell-Mediated Biodegradable Theranostic Nanoparticles for Melanoma Targeting and Drug Delivery. Small.

[11] Chen D., Dougherty C.A., Zhu K., Hong H. (2015). Theranostic applications of carbon nanomaterials in cancer: Focus on imaging and cargo delivery. J. Control. Release.

[12] Chen L., Zhong X., Yi X., Huang M., Ning P., Liu T., Ge C., Chai Z., Liu Z., Yang K. (2015). Radionuclide ^131^I labeled reduced graphene oxide for nuclear imaging guided combined radio- and photothermal therapy of cancer. Biomaterials.

[13] Zhang S., Yang K., Feng L., Liu Z. (2011). In vitro and in vivo behaviors of dextran functionalized graphene. Carbon N. Y..

[14] Jiang D.W., Peng C., Sun Y.H., Jia L.N., Li J.B., Zhang L. (2015). Study on technetium-99m labeling of graphene oxide nanosheets through click chemistry-99mTc labeling of graphene oxide nanosheets. Nucl. Sci. Tech..

[15] Vardharajula S., Ali S.Z., Tiwari P.M., Eroǧlu E., Vig K., Dennis V.A., Singh S.R. (2012). Functionalized carbon nanotubes: Biomedical applications. Int. J. Nanomed..

[16] Hartman K.B., Hamlin D.K., Wilbur D.S., Wilson L.J. (2007). 211AtCl@US-tube nanocapsules: A new concept in radiotherapeutic-agent design. Small.

[17] Hong S.Y., Tobias G., Al-Jamal K.T., Ballesteros B., Ali-Boucetta H., Lozano-Perez S., Nellist P.D., Sim R.B., Finucane C., Mather S.J. (2010). Filled and glycosylated carbon nanotubes for in vivo radioemitter localization and imaging. Nat. Mater..

[18] Qi W., Li Z., Bi J., Wang J., Wang J., Sun T., Guo Y., Wu W. (2012). Biodistribution of co-exposure to multi-walled carbon nanotubes and nanodiamonds in mice. Nanoscale Res. Lett..

[19] Rojas S., Gispert J.D., Martín R., Abad S., Menchón C., Pareto D., Víctor V.M., Álvaro M., García H., Herance J.R. (2011). Biodistribution of amino-functionalized diamond nanoparticles. in vivo studies based on ^18^F radionuclide emission. ACS Nano.

[20] Ruggiero A., Villa C.H., Holland J.P., Sprinkle S.R., May C., Lewis J.S., Scheinberg D.A., McDevitt M.R. (2010). Imaging and treating tumor vasculature with targeted radiolabeled carbon nanotubes. Int. J. Nanomed..

[21] Vaquero J.J., Kinahan P. (2015). Positron Emission Tomography: Current Challenges and Opportunities for Technological Advances in Clinical and Preclinical Imaging Systems. Annu. Rev. Biomed. Eng..

[22] Challapalli A., Aboagye E.O. (2016). Positron Emission Tomography Imaging of Tumor Cell Metabolism and Application to Therapy Response Monitoring. Front. Oncol..

[23] Blower P.J. (2015). A nuclear chocolate box: The periodic table of nuclear medicine. Dalt. Trans..

[24] Edem P.E., Jørgensen J.T., Nørregaard K., Rossin R., Yazdani A., Valliant J.F., Robillard M., Herth M.M., Kjaer A. (2020). Evaluation of a ^68^Ga-Labeled DOTA-Tetrazine as a PET Alternative to ^111^In-SPECT Pretargeted Imaging. Molecules.

[25] Jødal L., Roivainen A., Oikonen V., Jalkanen S., Hansen S.B., Afzelius P., Alstrup A.K.O., Nielsen O.L., Jensen S.B. (2019). Kinetic Modelling of [68Ga]Ga-DOTA-Siglec-9 in Porcine Osteomyelitis and Soft Tissue Infections. Molecules.

[26] Hofman M.S., Beauregard J.-M., Barber T.W., Neels O.C., Eu P., Hicks R.J. (2011). ^68^Ga PET/CT Ventilation-Perfusion Imaging for Pulmonary Embolism: A Pilot Study with Comparison to Conventional Scintigraphy. J. Nucl. Med..

[27] Kazakov A.G., Garashchenko B.L., Yakovlev R.Y., Vinokurov S.E., Kalmykov S.N., Myasoedov B.F. (2020). An experimental study of sorption/desorption of selected radionuclides on carbon nanomaterials: A quest for possible applications in future nuclear medicine. Diam. Relat. Mater..

[28] Sauvenier G., Duyckaerts G. (1957). Dosage polarographique du germanium dans des minerals et concentrés germanifères. Anal. Chim. Acta.

[29] Olthof M.G.L., Tryfonidou M.A., Dadsetan M., Dhert W.J.A., Yaszemski M.J., Kempen D.H.R., Lu L. (2018). In Vitro and In Vivo Correlation of Bone Morphogenetic Protein-2 Release Profiles from Complex Delivery Vehicles. Tissue Eng. Part C Methods.

[30] Shibata H. (2017). Fabrication and functionalization of inorganic materials using amphiphilic molecules. J. Oleo Sci..

[31] Jin W.G., Chen W., Xu P.H., Lin X.W., Huang X.C., Chen G.H., Lu F., Chen X.M. (2017). An Exceptionally Water Stable Metal–Organic Framework with Amide-Functionalized Cages: Selective CO_2_/CH_4_ Uptake and Removal of Antibiotics and Dyes from Water. Chem. A Eur. J..

[32] Cole L.E., McGinnity T.L., Irimata L.E., Vargo-Gogola T., Roeder R.K. (2018). Effects of bisphosphonate ligands and PEGylation on targeted delivery of gold nanoparticles for contrast-enhanced radiographic detection of breast microcalcifications. Acta Biomater..

[33] Jokerst J.V., Lobovkina T., Zare R.N., Gambhir S.S. (2011). Nanoparticle PEGylation for imaging and therapy. Nanomedicine.

